# Clinicopathological Profile of Muscle Diseases Presenting the Adult Population in Northern India: Preliminary Analysis in a Limited Resource Setting

**DOI:** 10.7759/cureus.60084

**Published:** 2024-05-11

**Authors:** Apoorva Agarwal, Amrita Ghosh Kar, Deepika Joshi, Harshanayana Harshanayana

**Affiliations:** 1 Pathology, Era's Lucknow Medical College and Hospital, Lucknow, IND; 2 Pathology, Institute of Medical Sciences, Banaras Hindu University, Varanasi, IND; 3 Neurology, Institute of Medical Sciences, Banaras Hindu University, Varanasi, IND

**Keywords:** muscular dystrophies, myopathy, neurological, neuropathy, muscle disease

## Abstract

Background: Muscle diseases are of various types, viz., muscular dystrophies, inflammatory myopathies, myotonic disorders, congenital myopathies, and metabolic myopathies. They all present with muscle weakness, be it proximal or distal. The assessment of muscle biopsy with the help of enzyme histochemistry, histopathological, and immunohistochemical methods is an essential component in the diagnosis of neuromuscular disorders. The authors outline brief data on muscle diseases prevalent in the North Indian region.

Methods: Muscle biopsy was done, and the biopsy was freshly frozen in liquid nitrogen and sections were taken on a cryostat. Slides were then stained with hematoxylin and eosin (H&E), modified Gomori trichome (MGT), nicotinamide adenine dinucleotide hydrogenase (NADH), and succinic dehydrogenase (SDH) stains. Further specific immunohistochemistry tests were also done.

Result: Out of n=16 cases, three cases were diagnosed as Becker’s muscular dystrophy, two cases were diagnosed as inflammatory myopathy, four cases were diagnosed as Facioscapulohumeral muscular dystrophy, and one each case of dysferlinopathy and alpha sarcoglycanopathy.

Conclusion: Muscle diseases can cause different levels of physical disability and thus it is important to diagnose at the appropriate time to ensure proper treatment.

## Introduction

Muscular dystrophies, inflammatory myopathies, myotonic disorders, congenital myopathies and metabolic myopathies are a few of the common examples of muscle diseases presenting as chronic proximal and symmetrical muscle weakness and distal weakness [1]. The assessment of muscle biopsy is important as it provides histopathological and immunohistochemical assessment of myopathic and neuropathic features along with the detection of sarcomeric proteins and material for further genetic studies [2].

As there is very little published epidemiological data on muscle disease in India compared to the world, its prevalence is difficult to tell. Much information has accumulated globally in the last two decades on muscle disorders through advanced laboratory techniques, especially genetic analysis, but these are as yet not freely available in India except in a few places. This has made the identification and classification of known diseases and new variants (based on immunochemistry and genetic analysis) less accurate particularly in developing countries like ours.

In the present work, an attempt was made to study the clinicopathological profile of the muscle diseases presented by the adult population in the university hospital of Northern India and to diagnose and categorize muscle diseases on the basis of the morphological features, enzyme histochemistry, histopathological and immunohistochemistry (IHC) of fresh frozen and paraffin-embedded muscle biopsy specimens on light microscopy in the patients.

## Materials and methods

The study was done in the Department of Pathology, Institute of Medical Sciences (IMS), Banaras Hindu University (BHU) Varanasi, after institutional ethical approval from October 2016 to September 2017. This prospective study was based on muscle biopsy specimens of the adult patients attending OPD of the Department of Neurology, Sir Sunder Lal Hospital, BHU, Varanasi, with complaints of neuromuscular weakness who were clinically suspected to have muscle disease and underwent muscle biopsy after having informed consent. Ethical approval was obtained from the university vide letter no. Dean/2015-16/EC/447 dated November 9, 2016.

Patient selection and data collection

Inclusion criteria comprised any kind of muscle weakness with or without facial muscle weakness involvement and/or any kind of muscle weakness with a positive family history of muscle disease and/or patients presenting with muscle weakness above 15 years of age. Patients presenting with congenital weakness of muscle were excluded. A detailed history (e.g., age of onset, gender, age at presentation, duration of illness, clinical complaints, family history of the disease) clinical and neurological examination, routine baseline investigation (including electro-myographic study), muscle biopsy (under full aseptic conditions from a muscle that was moderately involved, vastus lateralis and gastrocnemius), enzyme biochemistry (e.g., creatinine phosphokinase level, lactate dehydrogenase level), IHC and microscopic examination were done for all enrolled patients. 

Pathological evaluation and interpretation

A muscle biopsy was done in the Department of Neurology, IMS BHU, and then transferred immediately to the Department of Pathology, IMS BHU in a box between ice packs. A part of the tissue was prepared for cryosection to get fresh frozen sections. Another small part of the tissue was fixed in 10% formalin to be processed for routine histopathologic examination.

Slides having fresh frozen sections were taken out and were stained for hematoxylin and eosin (H&E), modified Gomori trichome (MGT), nicotinamide adenine dinucleotide hydrogenase (NADH), and succinic dehydrogenase (SDH). For IHC to be done the individual powdered antibodies (Leica Novocastra) were reconstituted with PBS following their respective dilution factor as mentioned in Table 1. Peroxidase-conjugated secondary antibody (BioGenex, Fremont, CA) was used.

**Table 1:** Dilution factor for individual antibodies

IHC	Company	Clone	Ig class	Dilution
Spectrin	Leica Novocastra liquid mouse monoclonal	RBC2/3D5	IgG2b	1:100
Alpha Sarcoglycan	Leica Novocastra liquid mouse monoclonal (adhalin)	Ad1/20A6	IgG1	1:100
Beta Sarcoglycan	Leica Novocastra liquid mouse monoclonal antibody	BSarc/5B1	IgG1	1:100
Gamma Sarcoglycan	Leica Novocastra lyophilized mouse monoclonal antibody	35DAG/21B5	IgG2b, Kappa	1:100
Delta Sarcoglycan	Leica Novocastra lyophilized mouse monoclonal antibody	Dsarc3/12C1	IgG2a	1:40
Dystrophin N terminus	Leica Novocastra anticorps monoclonal lyophilise de souris (DYS 3)	Dy10/12B2	IgG2a	1:20
Dystrophin C terminus	Leica Novocastra lyophilized mouse monoclonal antibody (DYS 2)	Dy8/6C5	IgG1	1:20
Dystrophin R terminus	Leica Novocastra lyophilized mouse monoclonal antibody (DYS 1)	Dy4/6D3	IgG2a	1:20
Dysferlin	Leica Novocastra lyophilized mouse monoclonal antibody (hamlet)	Ham1/7B6	IgG1	1:40

The muscle tissue kept in formalin was further processed for paraffin embedding and 4-5 μm sections were cut and stained for H&E stain and Masson’s trichrome (MAT). All the stained sections were evaluated under a light microscope by two observers and pathological changes of muscle biopsy were recorded.

To evaluate abnormalities, the structure and appearance of muscle fibers were analyzed in the following order: muscle fibers (evaluated for changes in fiber shape and size, fiber type and pattern, changes in sarcolemmal nuclei, for degeneration and regeneration, changes in fiber architecture and structural abnormalities and for deficiency of enzymes); endomysium, perimysium, and epimysium (evaluated for fibrosis and adipose tissue and cellular reactions) and blood vessels.

Interpretation of IHC

Membranous expression in the muscle fibers was recorded as a positive result or normal finding and absent staining was recorded as a negative result and indicated deficient protein.

## Results

The demographic and clinical data have been summarized in Tables 2, 3, respectively.

**Table 2:** Demographic data of patients (n=16)

Demographic parameters (n=16)	Values
Age	Mean – 23.93 ± 6.3 years
	Median – 23 years
	Range – 15-36 years
Age Groups	
11-20 years	N=4 (25%)
21-30 years	N=9 (56.25%)
31-40 years	N=3 (18.75%)
Sex	Male (n=12)
	Female (n=3)

**Table 3:** Clinical profile of patients (n=16)

Symptom Presentation	No. of Patients, Percentage
Difficulty in walking	n=13, 81.25%
Difficulty in standing up from a sitting position	n=10; 62.5%
Difficulty in lifting objects above the head	n=4, 25%
Difficulty in climbing up stairs	n=3, 18.75%
Difficulty in closing eyes and smiling indicating facial involvement and muscle pain	n=3, 18.75%
Calf enlargement	n=3, 18.75%
Deviation of face	n=2, 12.5%
Difficulty in holding chappal	n=2, 12.5%
Rash	n=1, 6.25%
Onset of disease	Mean Age - 19.93 ± 5.89 years
	Second Decade (11-20 years, n=10, 62.5%)
	Third Decade (21-30 years, n=6, 37.5%)
Duration of Illness	Mean - 4 ± 1.03 years
	Range – 2 – 6 years
Familial Inheritance	Yes (n=5, 31%)
Site of Onset	Proximal lower limb muscle weakness (n=11, 68.75%)
	Distal lower limb muscle weakness (n=2, 12.5%)
	Proximal upper limb muscle weakness (n=3, 18.75%)
Power of Muscle (Upper Limb)	
2/5	n=2, 12.5%
3/5	n=3, 18.75%
4/5	n=7, 43.75%
5/5	n=4, 25.0%
Power of Muscle (Lower Limb)	
2/5	n=2, 12.5%
3/5	n=8, 50.0%
4/5	n=5, 31.25%
5/5	n=1, 6.25%

Laboratory and biochemical data

EMG Findings were available for only five out of 16 cases and all of them had myopathic pattern on EMG. Creatine kinase level was estimated in only eight cases out of 16 and the level was raised above the normal value of 198 IU/L in each of the cases (range 295-2,008.4 IU/L). Lactate dehydrogenase level was available for seven cases out of 16 and was raised above normal value in each of the cases. 

Muscle biopsy pathologic findings

All n=16 cases underwent open muscle biopsy. Fresh tissue was available in 12 cases. Formalin-fixed tissue was available in four cases only. Table 4 shows the detailed microscopic features of 16 cases of H&E stain on both frozen and paraffin-embedded sections. Out of 16 cases, 14 were adequate and two cases revealed only fibro-collagenous tissue.

**Table 4:** Microscopic features on H&E (frozen and paraffin sections) FA: Fascicular architecture, MPC: Myophagocytosis, RF: Regenerating fibers, FI: Fatty infiltration, IN: Internal nuclei, PF: Perimysial fibrosis, EF: Endomysial fibrosis, +: present, -: absent

Case No.	FA	Variation in muscle fiber size	Hypertrophic fibers	Atrophic (round/ angulated)	Split fibers	MPC	RF	Necrosis	FI	IN	PF	EF	Inflammation	Others
1.	Preserved	Mild	+	Angulated	+	-	-	-	-	+	-	-	-	Multinucleated atrophic fibers
2.	Preserved	Moderate	+	Angulated	+	+	+	-	-	-	-	-	-	Multinucleated atrophic fibers
3.	Preserved	Mild	+	-	+	+	-	-	-	-	-	-	-	
4.	Preserved	Moderate	+	Angulated	+	-	-	-	-	-	-	-	-	
5.	Preserved	Marked	+	Round	-	+	+	-	+	+	+	-	-	
6.														Fibrocollagenous tissue
7.	Preserved	Moderate	+	Round	-	+	+	+	-	-	+	+	-	
8.	Preserved	Mild	-	Angulated	-	-	-	-	-	-	-	-	-	
9.	Preserved	Moderate	+	Round	-	+	+	-	-	-	-	+	+	Vasculitis
10.	Preserved	Marked	+	Round	-	+	-	-	-	-	-	+	+	Perifascicular atrophy
11.	Preserved	Marked	+	Round	-	-	-	+	+	-	+	+	-	
12.	Preserved	Moderate	+	Angulated	+	+	-	-	-	-	-	-	-	
13.	Preserved	Moderate	+	Round	-	-	-	-	-	-	-	-	+	Vasculitis
14.														Fibrocollagenous tissue
15.	Preserved	Mild	-	Round	-	-	-	-	-	-	-	-	-	
16.	Preserved	Mild	-	-	+	-	-	-	-	-	-	-	-	

Staining and enzyme histochemistry findings 

**Figure 1:**
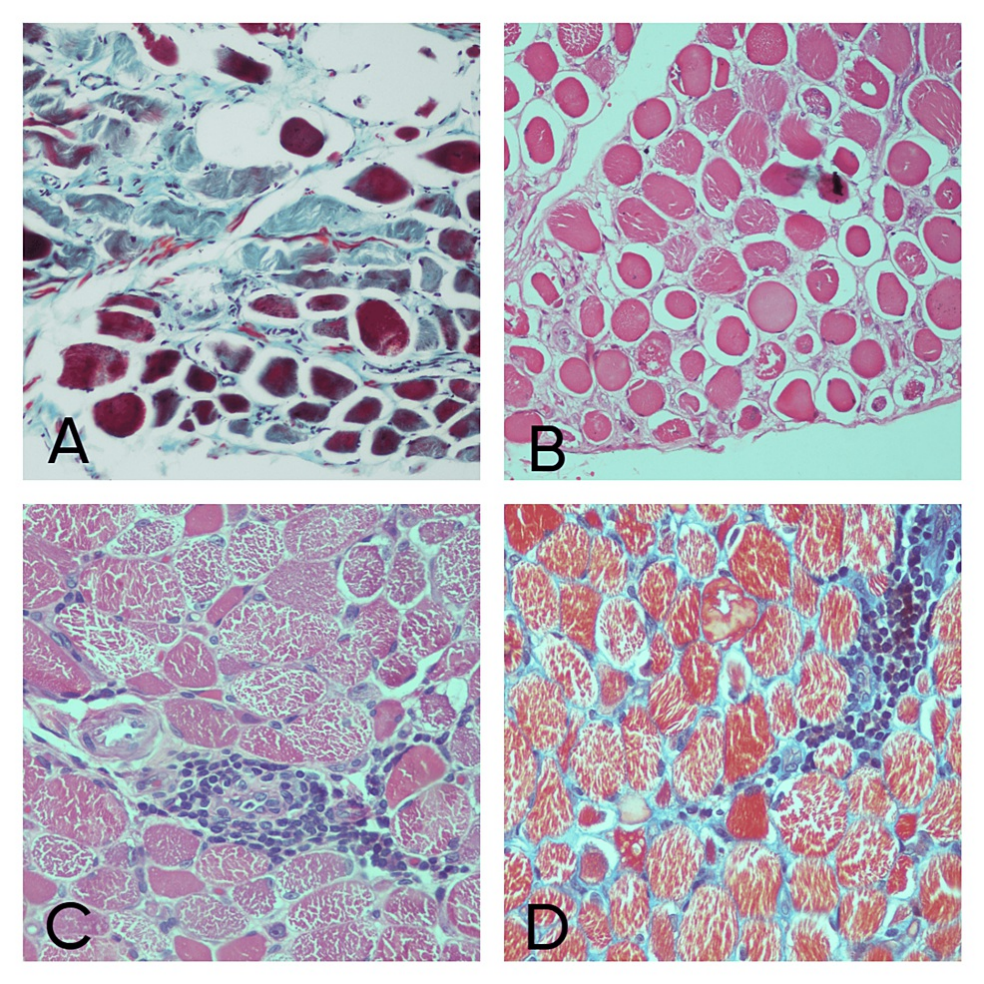
Inflammatory myopathy case: (A) MAT stain showing perimyseal fibrosis (x40), (B) H&E stain showing perifascicular atrophy (x20), (C) H&E stain showing perivascular inflammatory infiltrate (x40), (D) MAT stain showing perivascular inflammatory infiltrate (x40).

**Figure 2:**
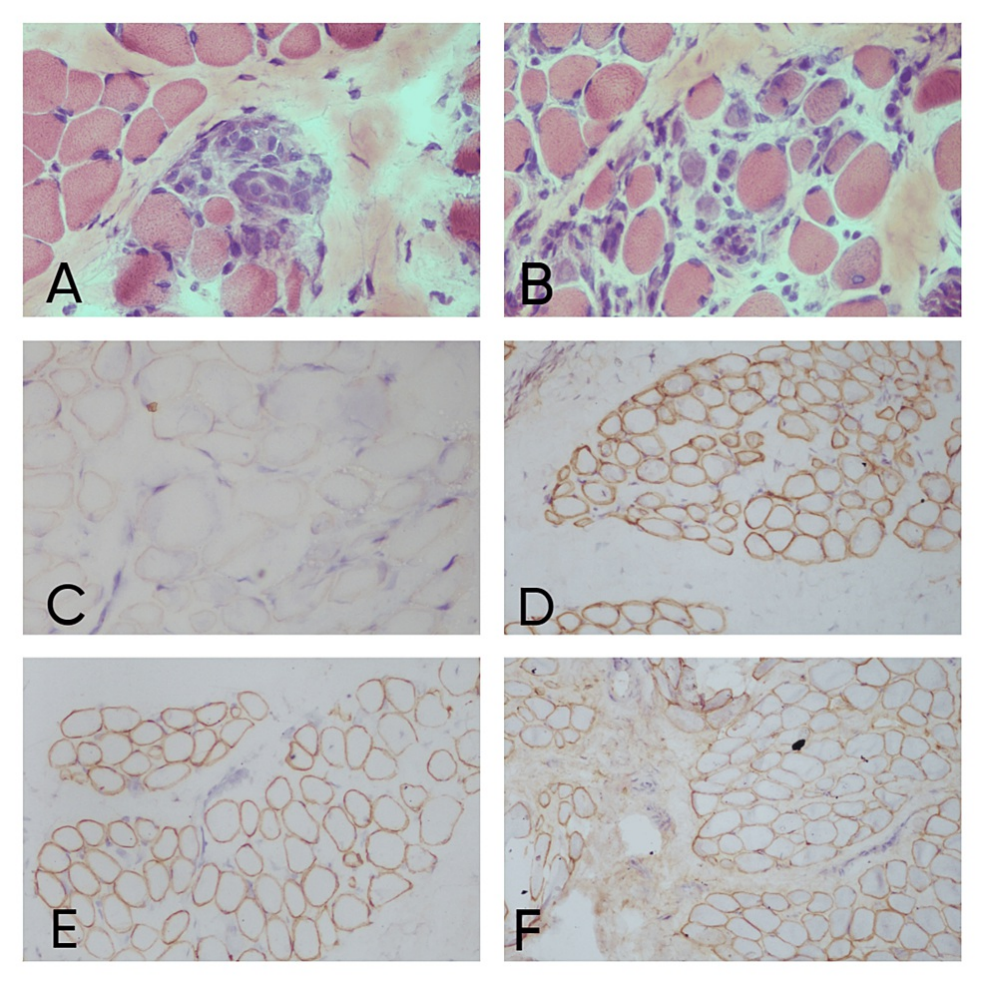
Becker’s muscular dystrophy case: (A) H&E stain showing myophagocytosis (x40), (B) H&E stain showing regenerating fibers (x40), (C) dystrophin N IHC stain showing mild positivity (x20), (D) alpha sarcoglycan IHC stain showing positivity (x20), (E) gamma sarcoglycan IHC stain showing positivity (x20), (F) Spectrin IHC stain showing positivity (x20).

An increase in fibrosis appeared as eosinophilic material on the H&E stain and was confirmed on the MAT stain where they appeared green in color. An increase in the connective tissue around each muscle fiber and leading to separation of the myofibrils indicated endomysial fibrosis (21.4%; n=3 cases) whereas an increase in the connective tissue around each muscle fascicle indicated perimysial fibrosis (28.5%; n=4 cases) (Figure 1A). Fat infiltration was seen in only 14.3% (n=2) cases. In one case perifascicular atrophy was present as was revealed by atrophy of myofibers, which formed the boundary of fascicles (Figure 1B). Other features like multinucleated atrophic fibers, internal nuclei and necrosis were seen in only 14.3% (n=2) cases each. Perivascular inflammation was present in 21.4% (n=3) cases and absent in 78.6% (n=11) cases (Figures 1C, 1D). Myophagocytosis was present in 50% (n=7) cases and absent in the same (Figure 2A). Regenerating fibers were present in 28.5% (n=4) cases and absent in 71.5% (n=10) cases (Figure 2B).

**Figure 3:**
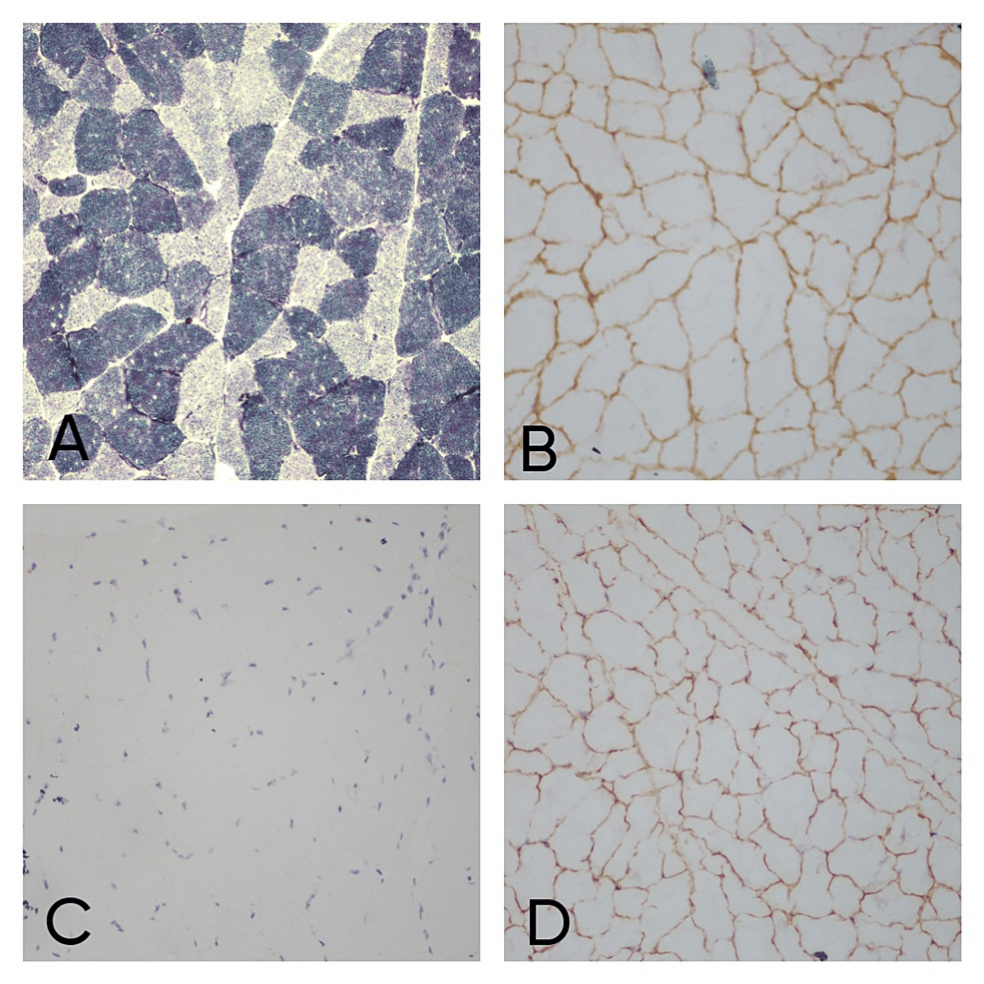
Dysferlinopathy case: (A) NADH staining showing Type I and Type II fibers (x20), (B) alpha sarcoglycan IHC stain showing positivity (x20), (C) dysferlin IHC stain showing negativity (x20), (D) dystrophin N IHC stain showing positivity (x20).

On MGT-stained sections of cytoplasm, myofibers appeared blue-green, nuclei appeared red and connective tissue green in all the cases. None of the cases revealed ragged red fibers, rimmed vacuoles, or any basophilic inclusions. On SDH stain & NADH stain, most of the cases revealed a checkerboard pattern of staining of type 1 fibers and type 2 fibers (Figure 3A). Type 1 fibers were stained darker than type 2 fibers. The stain also showed a punctate distribution throughout the cell because of predominant colocalization with mitochondria. In 14.2% (n=2) cases showed lobulated fibers and only one (7%) case showed moth-eaten fiber. No other abnormality findings on NADH and SDH stain-like target fibers, whorled and ring fibers and tubular aggregates were detected in our cases.

Based on the above observations on histopathological findings on H&E frozen sections, H&E paraffin sections, MAT, MGT, NADH, and SDH of the findings of muscle biopsies categories were categorized as - dystrophic pattern and inflammatory myopathy in 21.4% cases (n=3) each. Fifty percent (n=7) cases had myopathic pattern while one case showed no abnormality.

**Figure 4:**
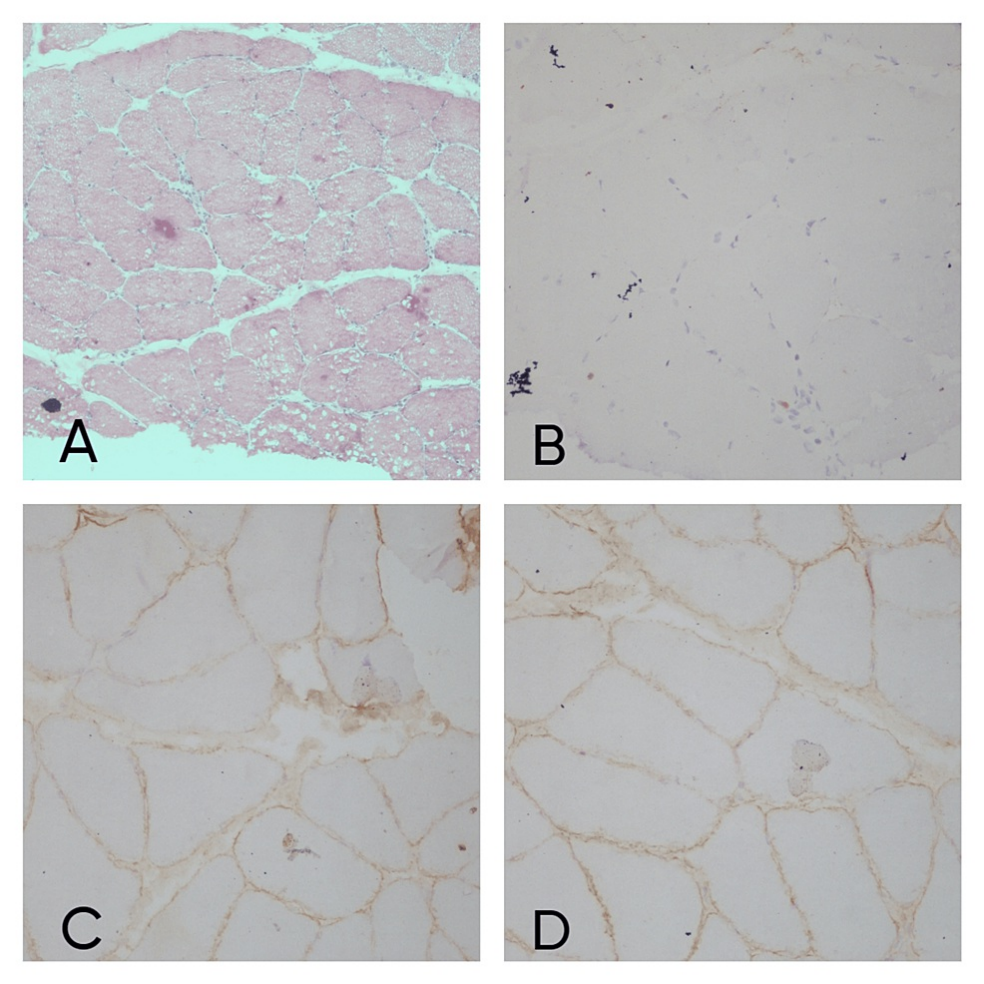
Alpha sarcoglycanopathy case: (A) H&E stain showing variation in muscle fiber (x10), (B) Alpha sarcoglycan IHC stain showing negativity (x20), (C) beta sarcoglycan IHC stain showing positivity (x20), (D) Gamma sarcoglycan IHC stain showing positivity (x20).

On IHC, out of 16 cases, three cases did not show positivity for dystrophin so they were labeled as Becker’s muscular dystrophy (BMD) (Figure 2C). One case was diagnosed as dysferlinopathy (Figure 3C) and alpha sarcoglycanopathy (Figure 4) as they did not show positivity for dysferlin and α sarcoglycan, respectively. Microscopic and immunohistochemical findings of 16 cases are elaborated in Table 5.

**Table 5:** Combining all clinical and pathological data to deduce a diagnosis AF: Atrophic Fibers, BMD: Becker’s Muscular Dystrophy, CPK: Creatine Phosphokinase, DM: Dermatomyositis, EF: Endomyseal Fibrosis, FA: Fascicular Architecture, FD: Final Diagnosis, FI: Fatty Infiltration, FSHD: Fascio Scapulo Humeral Disease, HF: Hypertrophic Fibers, IHC: Immunohistochemistry, IN: Internal Nuclei, LGMD: Limb Girdle Muscular Dystrophy, LL: Lower Limb, MPC: Myophagocytosis, N/A: Not Available, NAD: No Abnormality Detected, PD: Provisional Diagnosis, PF: Perimyseal Fibrosis, Pro: Proximal, RF: Regenerating Fibers, SF: Split Fibers, UL: Upper Limb

Case no	Age of cohort	Site of onset	Clinical features	CPK (IU/L)	Microscopic findings	IHC	PD	Final dDiagnosis
1	33	Prox, LL	difficulty in standing up from sitting position, lifting objects above head,	N/A	Mild variation in muscle fiber size, HF, angulated AF, internal nuclei		LGMD	Mild myopathy
2	20	Prox, UL	difficulty in raising hands above head and cycling, difficulty in closing eyes and smiling	N/A	Moderate variation in muscle fiber size, HF, angulated AF, MPC, SF, RF	All +ve	FSHD	FSHD
3	17	Prox, UL	difficulty in raising hands above head and deviation of face, closing eyes and smiling	707.8	Mild variation in muscle fiber size, HF, MPC, SF, lobulated fibers.	All +ve	FSHD	FSHD
4	22	Prox, LL	difficulty in standing up from sitting position, difficulty in climbing up the stairs	1089.7	Moderate variation in muscle fiber size, HF, angulated AF, SF	Dysferlin –ve, rest +ve	LGMD	Dysferlinopathy
5	14	Prox, LL	difficulty in walking, standing up from sitting position, calf hypertrophy	1845.4	Marked variation in muscle fiber size, HF, round AF, MPC, necrosis, FA, PF, RF, lobulated fibers.	Dystrophin –ve, rest +ve	BMD	BMD
6	28	Distal, LL	difficulty in holding chappal, walking, muscle pain	1606	Fibrocollagenous tissue only		distal myopathy/LGMD 2B	Inconclusive
7	17	Prox, LL	difficulty in walking, standing up from sitting position, thinning of quadriceps, calf hypertrophy	2008.4	Moderate variation in muscle fiber size, HF, round AF, MPC, necrosis, RF, PF & EF	Dystrophin mild positive, rest +ve	BMD	BMD
8	23	Prox, LL	difficulty in climbing up the stairs, difficulty in walking	N/A	Mild variation in muscle fiber size, angulated AF	All +ve	LGMD	NAD
9	36	Prox, LL	difficulty in standing up from sitting position, muscle pain, rash on face	N/A	Moderate Variation in muscle fiber size, HF, round AF, MPC, RF, EF and perivascular inflammation	All +ve	Inflammatory myopathy	Inflammatory myopathy associated with carcinoma
10	32	Prox, LL	difficulty in standing up from sitting position, muscle pain, rash on back	N/A	Marked Variation in muscle fiber size, HF, round AF, MPC, EF and perivascular inflammation	All +ve	inflammatory myopathy	Dermatomyositis
11	16	Prox, LL	bending of b/l knee during walking, thinning of quadriceps muscles, difficulty in standing up from sitting position	1504.5	Marked Variation in muscle fiber size, HF, round AF, necrosis, MPC	Dystrophin –ve, rest +ve	LGMD/BMD	BMD
12	22	Prox, LL	difficulty in standing up from sitting position, climbing up stairs, calf hypertrophy	295	Moderate Variation in muscle fiber size, HF, angulated AF, MPC, SF	Alpha –ve, rest +ve	LGMD/BMD	alpha
13	26	Prox, LL	difficulty in standing up from sitting position, rash on back	N/A	Moderate Variation in muscle fiber size, HF, round AF and perivascular inflammation	All +ve	inflammatory myopathy	Polymyositis
14	29	Prox, LL	difficulty in standing up from sitting position, walking	N/A	Fibrocollagenous tissue only		LGMD	Inconclusive
15	23	Distal, LL	difficulty in holding chappal, walking	N/A	Mild variation in muscle fiber size, round AF	All +ve	distal myopathy/LGMD 2B	myopathic features
16	24	Prox, UL	difficulty in raising hands above head and deviation of face, closing eyes and smiling	N/A	Mild Variation in muscle fiber size, HF, SF	All +ve	FSHD	FSHD

## Discussion

The literature available in India [3,4] underscores the routine occurrence of a broad spectrum of myopathies in the country. However, the limited availability of large-scale laboratory facilities, particularly in immunocytochemistry and genetic testing, hampers comprehensive molecular analysis of these myopathies. Consequently, the available data may not accurately reflect the true prevalence and characteristics of these conditions, as most published prevalence data stem from hospital series rather than population-based studies. In our present hospital-based study, which included 16 cases of adult-onset muscle weakness, many patients declined muscle biopsy due to a lack of awareness regarding its importance in diagnosing muscular dystrophy. Currently, only a few centers in India possess the necessary laboratory support to study various aspects of myopathies, resulting in incomplete analysis of existing cases. Age-based categorization is often absent in general population studies, leading to Duchenne muscular dystrophy (DMD) being commonly reported. Khadilkar et al. [5] noted DMD as the primary childhood myopathy, while limb-girdle dystrophy was predominant in adolescent and adult populations.

In our study, three cases were diagnosed as Fascio scapulo humeral disease (FSHD) out of 16 cases (18.275%). Srinivas et al. in 1975 [6] in a series of 211 cases of muscular dystrophy, reported that FSHD constituted only 2.3% incidence while Das et al. [3] in their study had an incidence of 1.3% of patients with FSHD phenotype. Khadilkar et al. in another work stated its incidence to be 2% to 3% of muscular dystrophy cases in India [7]. This incidence is notably higher compared to other series likely due to our study's focus on adult-onset muscle disease and its small sample size. All three FSHD cases in our study were male, consistent with Western literature, which often reports a male-to-female ratio of 1.5:1 [8,9]. Our study's mean age of symptom onset and disease presentation was comparable to previously published regional literature, suggesting consistency in findings across studies. In our study, one out of three cases had a familial inheritance history, as evidenced by the presence of similar clinical features in his sister. Published data has indicated a higher incidence of familial inheritance in FSHD, with percentages ranging from 60% to 100% [8,10]. All patients in our study exhibited upper limb weakness extending to the scapular region, resulting in difficulty in raising objects above the head. Additionally, facial deviation and difficulty in closing the eyes were noted, with one patient experiencing difficulty in cycling, indicating pelvic girdle weakness. The power of upper limb muscles was reduced, ranging from 2 to 3 out of 5. Krasnianski et al. [8] reported the onset of the disease primarily in facial or shoulder girdle muscles, with classical clinical features such as facial weakness, shoulder girdle muscle paresis, and scapular winging, followed by foot extensor weakness and later hip girdle paresis. Similarly, Tamhankar et al. [10] documented muscle weakness presentation in the majority of cases, including facial weakness, scapular winging, and pelvic girdle weakness. Greef et al. [9] also reported various incidences of muscle weakness, with scapular weakness being the most prevalent. Our study's clinical findings align with those reported in the literature. CPK levels were available for seven patients in our study, showing raised levels (mean - 680 U/L). Similar observations were reported by Krasnianski et al. [8] and Tamhankar et al. [10]. EMG findings in our study revealed a myopathic pattern, consistent with previous reports by Krasnianski et al. [8] and Tamhankar et al. [10]. Microscopic examination showed myopathic features, including variation in muscle fiber size, hypertrophic and atrophic fibers, and split fibers, in line with findings documented by Krasnianski et al. [8] and Statland et al. [11]. Enzyme histochemistry did not reveal significant findings, and IHC was negative for all markers tested, consistent with the limited availability of dedicated studies in FSHD cases. Additionally, we encountered three cases of BMD in our study, all male adolescents with two patients having a positive family history. Similar observations were reported by Kaido et al. [12], with a high incidence of family history in their study cohort. The mean age at onset in our study was comparable to previous findings by Emery et al. [13]. Clinical presentations in our study, including proximal muscle weakness and calf hypertrophy, aligned with descriptions in previous studies by Kaido et al. [12] and Emery et al. [13]. Furthermore, all cases in our study exhibited a proximal onset of lower limb weakness and elevated CPK levels, indicative of BMD.

In our study, microscopic examination revealed significant variability in muscle fiber size, characterized by the presence of round atrophic fibers, hypertrophic fibers, myophagocytosis, regenerating fibers, and increased connective tissue (fibrosis). Necrosis was identified in one case. Bonilla et al. [14] have documented similar microscopic findings, noting marked variations in fiber size, with a mixture of small and some hypertrophic fibers. They also mentioned fiber splitting, although regeneration and necrosis were infrequent occurrences. Likewise, Kaiso et al. [12] reported moderate to marked variation in fiber size in their study, with observations of both necrotic and basophilic regenerating fibers in most biopsies of BMD. They also noted the presence of central nuclei and mild to moderate interstitial fibrosis without notable fatty tissue infiltration. Split fibers were also identified, with 80% of patients exhibiting hypertrophic fibers and 35% displaying type 1 fiber atrophy in muscle biopsies.

In our IHC analysis, dystrophin N terminus exhibited weak positivity with membranous staining observed in some fibers and absence in others. However, dystrophin C and R terminus showed no membranous positivity. Conversely, all other markers (sarcoglycans, spectrin, dysferlin) displayed positive results. Currently, multiplex polymerase chain reactions (PCR) are widely employed in numerous centers across India to investigate the dystrophin gene, with comprehensive data available from major centers in the northern, western, and southern regions of the country [15-17]. Unfortunately, due to the unavailability of molecular analysis resources in our department, we were unable to corroborate our diagnosis using genetic tools.

Identifying forms of limb girdle muscular dystrophy (LGMD) with autosomal recessive inheritance presents a challenge due to numerous sporadic cases, inadequate familial history data, and its significant clinical overlap with Duchenne and Becker muscular dystrophies [18]. Prevalence estimates for all LGMD forms have varied widely, ranging from one per 14,500 to one per 123,000 populations. In our study, two cases (12%) were diagnosed with LGMD, specifically dysferlinopathy and α-sarcoglycanopathy, determined through IHC and clinical assessment. Srinivas et al. [6] reported an LGMD incidence of 16.5%, while Mondkar et al. [19] designated 12 cases (9.5%) as girdle dystrophy in their hospital-based study of 126 muscular dystrophy cases, with some not fitting the criteria for Duchenne or Becker muscular dystrophies. Similarly, Das et al. [3] identified LGMDs in 29.2% of cases, including severe dystrophies in young girls resembling DMD and autosomal recessive dystrophies in young boys. In a hospital-based survey at a neuromuscular clinic in Mumbai, approximately one-fourth of all patients were diagnosed with LGMD [5].

Due to our study's limited duration of only one year and its hospital-based nature, wherein we could only gather 16 cases, our findings might not fully represent the epidemiology of muscular diseases prevalent in northern India. Additionally, the diagnosis remained elusive in two cases due to constraints posed by the unavailability of specific antibodies for IHC, such as calpain-3, caveolin-3, TRIM32, FKRP, telethonin, titin, myotilin, filamin C, and lamin A/B, along with stains like COX and Acid phosphatase necessary for enzyme deficiency analysis crucial in studying metabolic disorders and mitochondrial myopathy. Moreover, the absence of genetic studies via PCR in our department posed a further limitation, impeding confirmation of diagnoses at the molecular level.

## Conclusions

We hereby conclude that as muscle disease can cause serious physical disability, it is necessary to diagnose it at the earliest so proper treatment can be given. As in our present study, a definitive diagnosis was made with the help of clinical history, histomorphological findings, enzyme histochemistry, and immunohistochemical findings. With the help of even IHC alone, different kinds of LGMDs can be categorized to a larger extent. Even in the absence of a clear pathologic diagnosis, reporting both positive and negative findings is essential to guiding the clinician’s additional testing toward achieving a diagnosis. Overall, our study emphasizes the need for enhanced laboratory facilities and comprehensive population-based studies to accurately assess the prevalence and characteristics of myopathies in India.

## References

[1] Barohn RJ, Dimachkie MM, Jackson CE (2014). A pattern recognition approach to patients with a suspected myopathy. Neurol Clin.

[2] Joyce NC, Oskarsson B, Jin LW (2012). Muscle biopsy evaluation in neuromuscular disorders. Phys Med Rehabil Clin N Am.

[3] Das S, Sarala D (1998). Diagnosis of muscular dystrophies : the changing concepts. Neurol India.

[4] Panigrahi I, Kesari A, Phadke SR, Mittal B (2002). Clinical and molecular diagnosis of spinal muscular atrophy. Neurol India.

[5] Khadilkar SV (2015). Limb girdle muscular dystrophies in India. Neurol India.

[6] Srinivas K (1975). The myopathies (1950-1975). Proc Inst Neurol.

[7] Khadilkar SV, Patil SG, Amin SN (2008). Study of idiopathic inflammatory myopathies with special reference to borderland between idiopathic inflammatory myopathies and muscular dystrophies. Neurol India.

[8] Krasnianski M, Neudecker S, Eger K, Jakubiczka S, Zierz S (2003). Typical facioscapulohumeral dystrophy phenotype in patients without FSHD 4q35 deletion. J Neurol.

[9] de Greef JC, Lemmers RJ, Camaño P (2010). Clinical features of facioscapulohumeral muscular dystrophy 2. Neurology.

[10] Tamhankar PM, Phadke SR (2010). Clinical profile and molecular diagnosis in patients of facioscapulohumeral dystrophy from Indian subcontinent. Neurol India.

[11] Statland JM, Barohn RJ, McVey AL, Katz JS, Dimachkie MM (2015). Patterns of weakness, classification of Motor neuron disease, and Clinical diagnosis of sporadic amyotrophic lateral sclerosis. Neurol Clin.

[12] Kaido M, Arahata K, Hoffman EP, Nonaka I, Sugita H (1991). Muscle histology in Becker muscular dystrophy. Muscle Nerve.

[13] Emery AE Population frequencies of inherited neuromuscular diseases--a world survey. Neuromuscul Disord.

[14] Bonilla E, Samitt CE, Miranda AF (1988). Duchenne muscular dystrophy: deficiency of dystrophin at the muscle cell surface. Cell.

[15] Dastur RS, Gaitonde PS, Khadilkar SV, Nadkarni JJ (2008). Becker muscular dystrophy in Indian patients: analysis of dystrophin gene deletion patterns. Neurol India.

[16] Mital A, Kumari D, Gupta M, Goyle S (1998). Molecular characterisation of Duchenne muscular dystrophy and phenotypic correlation. J Neurol Sci.

[17] Sinha S, Pradhan S, Mittal RD, Mittal B (1992). Detection of gene deletion in patients of Duchenne muscular dystrophy/Becker muscular dystrophy using polymerase chain reaction. Indian J Med Res.

[18] Dinçer P, Leturcq F, Richard I (1997). A biochemical, genetic, and clinical survey of autosomal recessive limb girdle muscular dystrophies in Turkey. Ann Neurol.

[19] Mondkar VP, Bhabha SK (1984). Muscular dystrophy (a clinical analysis of 126 cases). J Postgrad Med.

